# *Advenella alkanexedens*, a specific phosphate-solubilizing bacterium from rapeseed rhizosphere soil, highly activates insoluble phosphorus in calcareous soil

**DOI:** 10.1128/spectrum.03481-25

**Published:** 2026-05-26

**Authors:** Yani Hao, Wenping Yang, Jingjing Chai, Hongyu Jiang, Shihang Hao, Haotian Luo, Guangsheng Zhou, Tingdong Fu, Zhiqiang Gao, Zhenping Yang

**Affiliations:** 1 College of Agriculture/Collaborative Innovation Center for High-quality and Efficient Production of Characteristic Crops on the Loess Plateau, Shanxi Agricultural University 644568, Taigu, Shanxi, China; 2 College of Life Sciences, North China University of Science and Technology 128790, Tangshan, Hebei, China; 3 Faculty of Business, Economics and Law, The University of Queensland 1974, Brisbane, Queensland, Australia; 4 Science Faculty, The University of Queensland, Brisbane, Queensland, Australia; 5 College of Plant Sciences & Technology, Huazhong Agricultural University 47895, Wuhan, Hubei, China; Connecticut Agricultural Experiment Station 5758, New Haven, Connecticut, USA

**Keywords:** *Advenella alkanexedens*, soil phosphorus activation, inorganic phosphorus components, phosphate-solubilizing microbe, whole targeted metabolome

## Abstract

Phosphorus fixation represents a primary constraint limiting the agronomic efficiency of phosphate fertilizers in calcareous soil. Rapeseed roots secrete amounts of organic matter, which can mobilize and decompose insoluble phosphorus in the soil. However, the activation mechanism of sparingly insoluble phosphorus in calcareous soils by rapeseed rhizosphere microorganisms remains unclear. This study aimed to screen and identify phosphate-solubilizing microorganisms from the rapeseed rhizosphere of calcareous soil, and to elucidate their key metabolic pathways for activating insoluble phosphorus. The results demonstrated that (i) fourteen dominant phosphate-solubilizing strains were isolated from rapeseed rhizosphere soil. Among these, *Advenella alkanexedens* was verified to significantly promote wheat growth and increase soil available phosphorus content. (ii) The culture condition optimization and functional characterization for *Advenella alkanexedens* revealed that its optimal growth temperature was 30°C, with an initial pH of 7. Its phosphate-solubilizing ability was regulated by Mg^2+^, K^2+^, and Ca^2+^ ions, and the strain exhibited considerable salt tolerance and the ability to produce siderophores. (iii) *Advenella alkanexedens* increased soil available phosphorus content by 11.49%–81.91% and elevated phytase activity by 36.87%–82.49%. Correlation analysis indicated that soil available phosphorus and phytase activity were significantly positively correlated with Ca_2_–P, Ca_8_–P, and Al–P fractions. (iv) Amino acids and organic acids were identified as the key metabolites influencing the phosphate-solubilizing function of *Advenella alkanexedens*. The KEGG pathway analysis showed these metabolites were primarily enriched in β-alanine metabolism and arginine and proline metabolism pathways. Our findings confirm that *Advenella alkanexedens* not only promotes crop growth but also significantly increases labile P fractions (Ca_2_–P, Ca_8_–P, Al–P) while reducing more stable forms (Ca_10_–P), thereby enhancing soil phosphorus use efficiency. This study holds important implications for planting rapeseed to activate insoluble phosphorus in soil, to reduce phosphate fertilizer application, and to promote sustainable utilization of soil phosphorus resources. Furthermore, it provides a theoretical foundation for developing agricultural microbial inoculants.

**IMPORTANCE:**

Our results confirm that *Advenella alkanexedens* not only benefits crop growth but also converts insoluble phosphates (O-P, Ca10-P) into highly active inorganic phosphorus components, thereby enhancing the utilization efficiency of soil phosphorus. This study was of great significance in activating the insoluble phosphorus in the soil, reducing the input of phosphate fertilizers, achieving the sustainable utilization of phosphorus resources, and protecting the environment. Additionally, it provided a basis for developing agricultural microbial agents.

## INTRODUCTION

Phosphorus (P) ranks as the second most essential nutrient for plant growth and development, playing a critical role in numerous physiological and biochemical processes, such as nutrient uptake, biological oxidation, and energy metabolism (1). Although P is generally abundant in soils, the majority exists in insoluble forms that are largely unavailable to plants (2). It is estimated that only 10%–25% of soil phosphorus is directly absorbed and utilized by crops during a growing season (3), indicating that the phosphorus in the soil cannot meet the high-yield demands of crops (4). Consequently, the application of inorganic phosphorus fertilizers is considered essential to maintain high crop productivity. However, excessive use of chemical P fertilizers not only reduces phosphorus use efficiency (5) but also promotes the accumulation of insoluble phosphorus in soils (6). Therefore, enhancing phosphorus solubility in the soil solution and mitigating phosphorus fixation are crucial steps toward sustainable nutrient management (7).

Soil phosphorus exists primarily in organic and inorganic forms, with the inorganic fraction dominating and typically accounting for 60% to 80% of total phosphorus (8). The various forms of inorganic phosphorus differ considerably in their transformation rates and availability to plants and microorganisms (9). Only water-soluble inorganic phosphorus—mainly H_2_PO_4_^−^ and HPO_4_^2−^—is directly accessible for plant uptake (10). Based on mineral binding characteristics and plant availability, inorganic soil phosphorus can be classified into three pools: (i) labile phosphorus, which includes dicalcium phosphate (Ca_2_–P); (ii) moderately-occluded phosphorus, comprising octacalcium phosphate (Ca_8_–P), variscite (Al–P), and strengite (Fe–P); and (iii) recalcitrant phosphorus, consisting of occluded P (O–P) and hydroxylapatite (Ca_10_–P) (11). Among these, Ca_2_–P is readily available to plants, while Ca_8_–P and Al–P can be transformed into Ca_2_–P under suitable conditions (12). These phosphorus fractions participate in diverse biogeochemical cycles and maintain a dynamic equilibrium, wherein transformations and constraints are interdependent. Consequently, the specific forms of phosphorus present in soil fundamentally govern its bioavailability and effectiveness (13).

Microorganisms play a pivotal role in the speciation and transformation of soil phosphorus, facilitating the conversion of organic to inorganic forms and driving the soil phosphorus cycle. Through mineralization mediated by soil microbes and enzymes, organic phosphorus is converted into inorganic phosphorus, while desorption and dissolution processes render inorganic phosphorus bioavailable (14). Phosphate-solubilizing microorganisms (PSM) comprise a diverse group of organisms—including actinobacteria, bacteria, fungi, arbuscular mycorrhizae, and cyanobacteria—that hydrolyze both organic and inorganic phosphorus into soluble forms, thereby increasing phosphorus bioavailability for plants (15). Among soil microbes, phosphate-solubilizing bacteria (PSB) represent 1%–50% of the microbial community, while phosphate-solubilizing fungi account for only 0.1%–0.5% (16). The microbial mobilization of inorganic phosphorus primarily occurs through the production and secretion of organic acids, inorganic acids, siderophore exopolysaccharides, and proton extrusion (17, 18). Notably, organic acids, such as citric and malic acids, secreted by rapeseed roots, are key agents in activating insoluble soil phosphorus. Furthermore, acid phosphatase present in root exudates also contributes significantly to this activation process (19).

The soils in the Jinzhong region are predominantly calcareous cinnamon soils, characterized by high calcium content and notably low levels of available phosphorus. To improve phosphorus bioavailability in such soils, inoculation with highly efficient PSMs represents one of the most promising strategies. In this study, a single PSM strain was isolated from the rapeseed rhizosphere soil. Previous research on this strain has mainly focused on its role in regulating intestinal flora (20) and its application in environmental bioremediation through pollutant degradation (21). However, the phosphate-solubilizing characteristics of *Advenella alkanexedens* and its involvement in phosphorus speciation and transformation in soil remain largely unexplored and warrant further investigation. This study aims to elucidate the phosphate-solubilizing metabolic mechanisms of this strain, providing a theoretical basis for the efficient utilization of soil phosphorus and establishing a foundation for its future field application.

## MATERIALS AND METHODS

### Isolation and screening of phosphate-solubilizing microorganisms

#### Culture medium used

Potato-saccharose-agar medium (PSA): potato 200 g, agar 20 g, sucrose 20 g, use distilled water to make up the volume to 1,000 mL.

Beef extract peptone AGAR medium: beef paste 3 g, peptone 10 g, NaCl 5 g, agar 20 g, NaOH 0.2 g, use distilled water to make up the volume to 1,000 mL.

National Botanical Research Institute’s phosphate (NBRIP) medium: dextrose 10 g, (NH_4_)_2_SO_4_ 0.1 g, KCl 0.2 g, MgCl_2_ 5 g, MgSO_4_·7H_2_O 0.25 g, Ca_3_(PO_4_)_2_ 5 g, agar 20 g.

#### Isolation and screening

Isolation: culturable microorganisms were isolated from fresh rapeseed rhizosphere soil samples using a gradient dilution plating method. Serial dilutions (10^−1^ to 10^−7^) of a soil suspension were prepared. Fungal isolates were obtained by plating the 10^−1^ to 10^−3^ dilutions on PSA medium. Bacterial isolates were obtained by plating the 10^−5^ to 10^−7^ dilutions on beef extract peptone agar medium (22).

Screening for phosphate-solubilizing activity was performed using the isolated colonies on NBRIP agar medium. Aliquots (15 mL) of the medium were poured into 90 mm Petri dishes. After solidification, distinct colonies were spot-inoculated, and plates were incubated at 26°C for 7 days (triplicate). Colonies forming a solubilization halo were considered positive. These putative PSMs were cultured in liquid NBRIP medium at 28°C, 160 rpm for 72 h. The soluble phosphorus in the cell-free supernatant (centrifuged) was quantified by molybdenum-antimony colorimetry, with sterile medium as the blank (23).

#### Genomic insights into the taxonomy of *Advenella alkanexedens*

Genomic DNA was extracted following the method described by Gao et al. (24). The quality of the extracted DNA was assessed by 1% agarose gel electrophoresis, and DNA samples that passed quality control were used for 16S rRNA gene amplification.

The universal bacterial primers 27F and 1492R were used to amplify the 16S rRNA gene sequence of *Advenella alkanexedens* with genomic DNA as the template. The amplification products were verified by 1% agarose gel electrophoresis and subsequently sent to Shanghai Personalbio Technology Co., Ltd. for routine sequencing.

The obtained sequences were queried against the NCBI database. The 16S rRNA sequence of *Advenella alkanexedens* was aligned with that of the type strain, and relevant sequences from closely related species were selected for homology analysis. A neighbor-joining (NJ) phylogenetic tree was constructed using Mega 5.0 to evaluate the taxonomic status of the strain from a molecular perspective.

### Optimization of culture conditions for *Advenella alkanexedens*

*Advenella alkanexedens* bacterial culture was inoculated at 1% (vol/vol) into 100 mL of NBRIP medium and incubated with shaking at five different temperatures: 20°C, 25°C, 30°C, 35°C, and 40°C. To assess the effect of pH, the medium was adjusted to initial pH values of 4, 5, 6, 7, 8, and 9 using 1 mol/L HCl or NaOH, while keeping all other medium components unchanged. For salinity tolerance tests, NaCl was added to achieve final mass percentages of 0%, 1.0%, 2.0%, 3.0%, 4.0%, 5.0%, 6.0%, and 7.0% in the NBRIP medium without altering other constituents. To evaluate the influence of metal ions, Cu, Mg, K, Al, Mn, and Ca were separately added at 1% (wt/vol) to 100 mL of NBRIP medium containing 1% (vol/vol) bacterial inoculum. All treatments were performed in triplicate, with a non-inoculated medium serving as the blank control. After incubation at 28°C and 160 rpm for 72 h, the soluble phosphorus content in the fermentation broth was determined.

Phosphate solubilization rate (%) = (Soluble phosphorus content in inoculated treatment − Soluble phosphorus content in control)/Amount of inorganic phosphorus source added × 100%.

### Pot experiment

#### Soil tested

The pot experiment was conducted at the experimental station of Shanxi Agricultural University, located in the Taigu District, Shanxi Province, China (112°28′E, 37°12′N). This area belongs to the late-maturing winter wheat zone of northern China and lies on the eastern margin of the Loess Plateau. Calcareous soils collected from a depth of more than 2 m were used in the experiment, with the following basic properties: organic matter 12.57 g/kg, available phosphorus 8.07 mg/kg, total phosphorus 0.58 g/kg, total nitrogen 0.89 g/kg, and pH 8.17.

#### Wheat variety and chemical fertilizers

The wheat variety used in this study was Changmai 6794, a high-water-use efficiency cultivar developed by Shanxi Agricultural University in China.

The chemical fertilizers tested were urea (*N* ≥ 46%) as the nitrogen source, calcium superphosphate (P_2_O_5_ ≥ 16%) as the phosphorus source, and potassium chloride (K_2_O ≥ 60%) as the potassium source.

#### Experimental design

Seeds of wheat (*Triticum aestivum* L.) were surface-sterilized in 5% NaClO for 5 min, rinsed thoroughly with distilled water, and blot-dried. Air-dried soil was passed through a sieve and added to pots (10 cm height × 15 cm diameter), with each pot containing 1.5 kg of soil. A basal dose of nitrogen (0.48 g as urea) and potassium (0.42 g) was thoroughly mixed into the soil of all pots prior to planting. The pots were then assigned to three treatment groups: (i) B4: soil amended with the fermentation broth of *Advenella alkanexedens* only; (ii) CK (positive control): soil uniformly blended with 0.45 g of phosphorus (as calcium superphosphate); (iii) CK-P (negative control): soil receiving no phosphorus. All pots were watered equally after sowing. At the seedling stage, six uniform seedlings were retained per pot. *Advenella alkanexedens* was prepared as a bacterial suspension (10^6^ CFU/mL), and 100 mL was applied around the roots of wheat plants in the B4 treatment group. For the control groups (CK and CK-P), an equal volume of distilled water was applied instead of the bacterial suspension. Each treatment was replicated four times, resulting in a total of 12 pots.

#### Colony-forming unit quantification for inoculum

The bacterial concentration was determined using the standard plate colony counting method. The suspension was serially diluted 10-fold to obtain gradients from 10^−1^ to 10^−9^. Then, 0.1 mL aliquots from dilutions of 10^−4^ to 10^−8^ were spread onto Luria-Bertani agar plates. After incubation at 37°C for 24 h, the number of visible colonies was counted, and the colony-forming unit (CFU) concentration of the original suspension was calculated and expressed as CFU·mL^−1^.

#### Sampling

Soil samples were collected from the wheat root zone of each pot using a sterilized soil auger. After removing visible plant residues and stones, the soils were passed through a sterile 1-mm sieve. Soils from replicate pots of the same treatments were pooled to form one composite sample. Each composite sample was then divided into three subsamples, placed in separate self-sealing bags, and promptly transported to the laboratory for analysis of soil physicochemical properties.

#### Indexes measurement

##### Soil physicochemical properties

Soil total phosphorus concentration was determined by digestion with H_2_SO_4_–HClO_4_ (25). Available phosphorus was extracted with the 0.5-mol/L NaHCO_3_ and measured using the molybdenum-antimony anti-spectrophotometric method (25). Phosphatase activity was assessed by sodium phenylphosphate colorimetry (25). Soil organic matter content was determined by the potassium dichromate volumetric method (25). Phytase activity was measured following a spectrophotometric procedure (26). For soil pH and electrical conductivity, air-dried soil (10 g, <2 mm) was mixed with deionized water at a ratio of 1:5 (wt/wt), shaken for 15 min, allowed to settle for 30 min, and then measured using a pH electrode and a conductivity meter, respectively.

Soil inorganic phosphorus fractions were separated and quantified using the sequential extraction procedure described by Jiang et al. (27). Six inorganic P fractions—dicalcium phosphate (Ca_2_–P), octacalcium phosphate (Ca_8_–P), aluminum phosphate (Al–P), iron phosphate (Fe–P), occluded phosphorus (O–P), and decacalcium phosphate (Ca_10_–P) were sequentially extracted with the following solutions in order: 0.25 mol/L NaHCO_3_ (pH 7.5), 0.5 mol/L NH_4_OAc (pH 4.2), 0.5 mol/L NH_4_F (pH 8.2), 0.8 mol/L H_3_BO_3_, 0.1 mol/L NaOH and Na_2_CO_3_ mixture, 0.3 mol/L sodium citrate (C_6_H_5_Na_3_O_7_) containing 1 g Na_2_S_2_O_4_ and 0.5 mol/L NaOH, and finally 0.5 mol/L H_2_SO_4_.

##### The whole targeted metabolomics analysis

For metabolite analysis, the phosphate-solubilizing strain was cultured to prepare a fermentation broth. A non-inoculated medium was included as the control, with six replicates per treatment. Sample preparation followed the protocol described by Demurtas et al. (28). Liquid chromatography conditions were set according to Zelena et al. (29), and mass spectrometry parameters were adopted from Want et al. (30).

### Statistical analyses

Statistical analyses were performed with SPSS Statistics 26.0 (China). For all statistical analyses and regressions, data were tested for normality (Shapiro-Wilk) and homogeneity of variance (Brown-Forsythe). Differences among treatments were compared by one-way ANOVA, followed by LSD post hoc tests (α = 0.05). Figures were generated with Origin 2022. Partial least squares-discriminant analysis (PLS-DA) was conducted using the R package Ropls (31), with a permutation test to evaluate overfitting. Potential biomarkers were identified based on variable importance in projection >1 and *P* value < 0.05, along with fold change between groups. Differential metabolites were subsequently subjected to functional enrichment and topological analysis using MetaboAnalyst (32), and relevant pathways were visualized via KEGG Mapper.

## RESULTS

### Isolation and screening of the dominant phosphate-solubilizing strains

**Fig 1:**
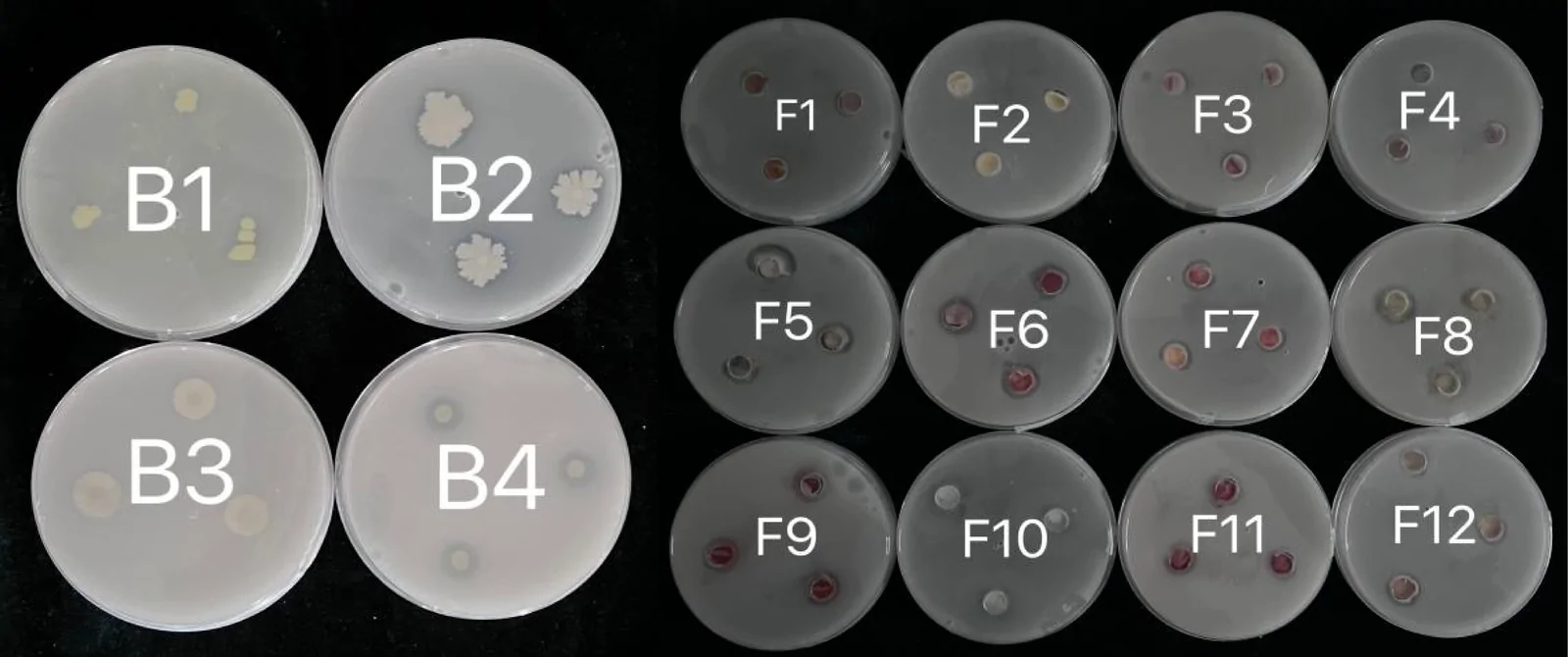
Phosphate-solubilizing microorganisms can be cultivated in the rhizosphere soil of rapeseed; (B) bacteria, (F) fungi.

Using culturable microorganisms isolated from rapeseed rhizosphere soil as the screening source (Fig. S1), we performed the screening on NBRIP solid medium, which allows the formation of distinct phosphate-solubilizing halos. On this medium, 4 bacterial and 12 fungal species were observed to produce clear phosphate-solubilizing zones. The appearance of such halos indicates that these strains likely secrete compounds capable of solubilizing the calcium phosphate present around the colonies. Based on this activity, 16 strains were initially selected as putative phosphate solubilizers (Fig. 1). Subsequent fermenting in an inorganic phosphorus liquid medium and measurement of soluble phosphorus content confirmed the phosphate-solubilizing ability in 13 of these strains. The strain numbers and species identities are listed in Table 1. These strains were then inoculated into pot-grown wheat to further validate their phosphate-solubilizing performance (see Table S2 and S3). Through principal component analysis, one bacterial strain, designated B4, was finally identified as exhibiting the strongest phosphate-solubilizing capacity (Table S4).

**TABLE 1:** Sequence identification of soil phosphorus soluble microorganisms and determination of phosphorus soluble ability^*a*^

Number	DNA identification results	Soluble phosphorus content (mg/L)
B1	*Pseudarthrobacter oxydans*	–^*b*^
B2	*Priestia aryabhattai*	4.03 ± 0.13bcde
B3	*Microbacterium hydrocarbonoxydans*	0.96 ± 0.02f
B4	*Advenella alkanexedens*	5.52 ± 0.74abc
F1	*Fusarium oxysporum*	0.22 ± 0.01f
F2	*Aspergillus neoniger*	6.20 ± 1.07ab
F3	*Ascomycota* sp.	6.29 ± 0.81a
F4	*Rhizopus oryzae*	3.09 ± 0.26de
F5	*Ascomycota* sp.	3.87 ± 0.34cde
F6	*Ascomycota* sp.	5.76 ± 0.73abc
F7	*Ascomycota* sp.	5.55 ± 1.02abc
F8	*Penicillium oxalicum*	5.14 ± 0.96abcd
F9	*Talaromyces flavus*	1.97 ± 0.02ef
F10	*Trichoderma viride*	6.86 ± 0.11a

^*a*^ Different lowercase letters in the same column indicate significant differences between treatments (*P *< 0.05).

^*b*^ –, not detected.

### *Advenella alkanexedens:* strain identification and optimization of its phosphate-solubilizing conditions

**Fig 2:**
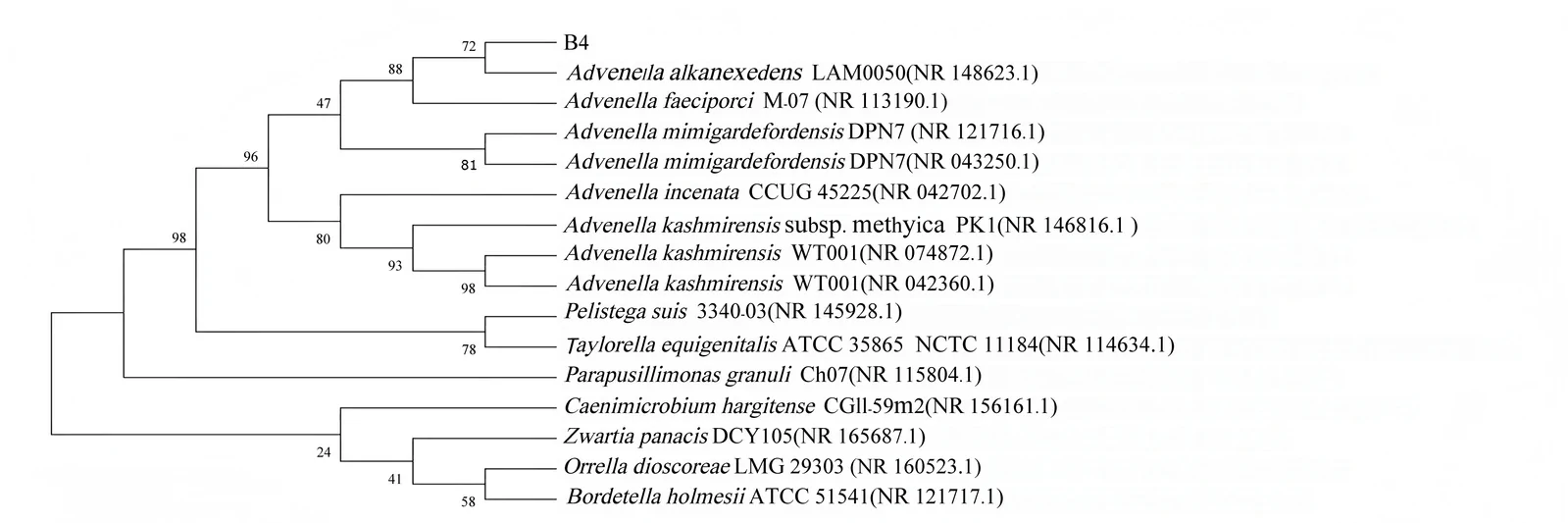
The NJ phylogenetic tree of phosphate-solubilizing bacterium B4 and its closely related species based on 16S rRNA sequences.

Based on PCR amplification and sequencing, an 872 bp fragment of the 16S rRNA gene was obtained from the phosphate-solubilizing strain B4. Sequence comparison with type strains revealed that the 16S rRNA sequence of strain B4 showed 72% bootstrap support with that of *Advenella alkanexedens* strain LAM0050 (NR_148623.1), a representative member of the genus Advenella. Phylogenetic analysis further confirmed that strain B4 forms a stable clade with this reference strain (Fig. 2). Combined with the morphological characteristics of strain B4 (Fig. 1), the 16S rRNA sequence data support the identification of strain B4 as *Advenella alkanexedens*, which has been deposited in NCBI under accession number PV576239.

*Advenella alkanexedens* is a gram-negative bacterium. On solid medium, its colonies appear yellowish-brown, smooth, moist, and semi-transparent. They are small in size, with regular margins, a raised center, and no halo formation. Functional characterization confirmed that this strain exhibits the ability to produce siderophores and solubilize inorganic phosphorus (Table S1).

**Fig 3:**
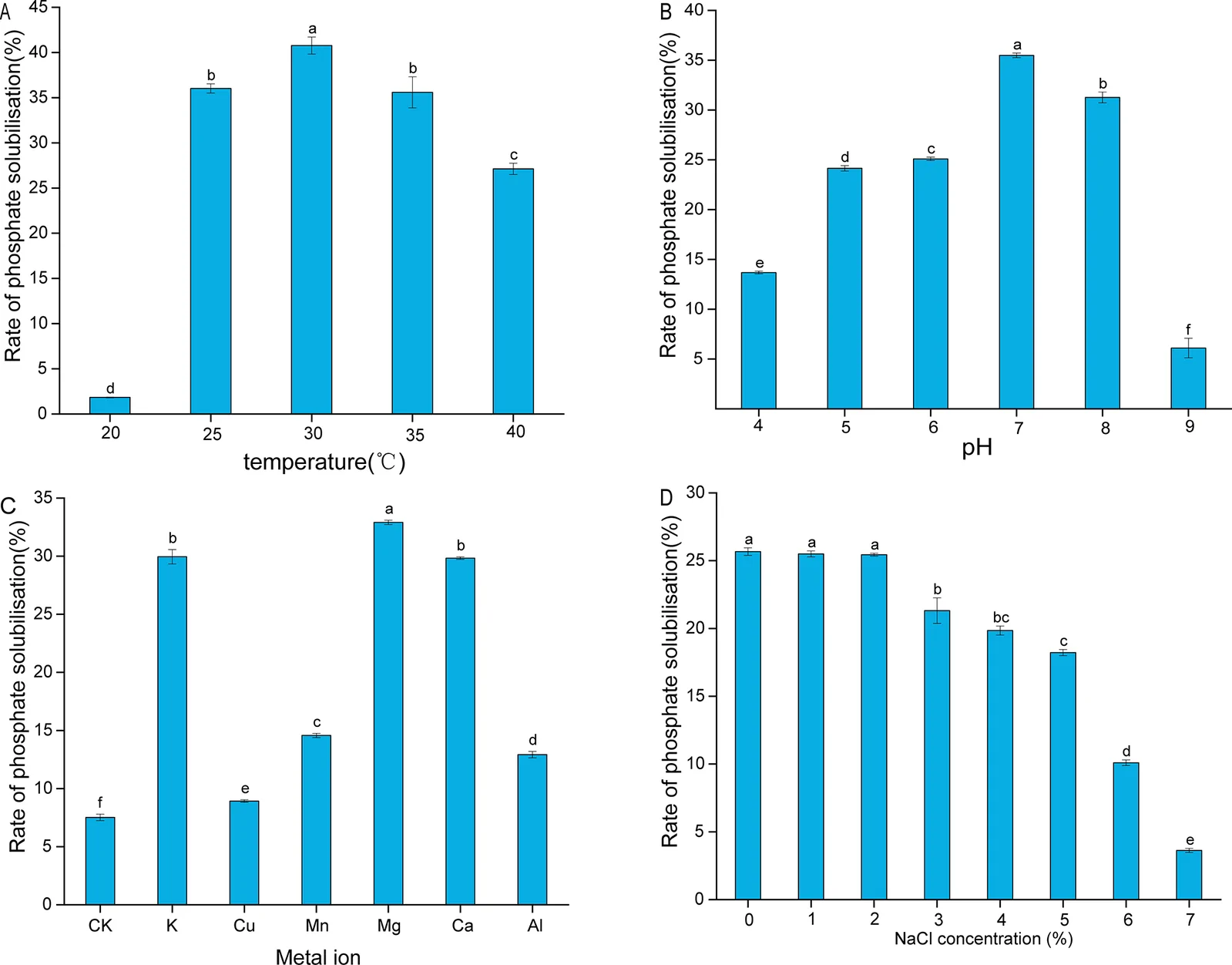
The effect of environmental factors ([**A**] temperature, [**B**] pH, [**C**] metal ion, and [**D**] NaCI concentration) on the phosphate-solubilizing ability of *Advenella alkanexedens*. Different lowercase letters indicate significant differences before and after returning to soil in different treatments (*P* < 0.05).

Temperature exerted a pronounced influence on the phosphate-solubilizing ability of strain B4 (Fig. 3A). The phosphate-solubilizing capacity increased initially with rising temperature, peaking at 30°C with a solubilization rate of 40.78%, and then declined. At pH 7, the maximum phosphate solubilization was observed, corresponding to a solubilization rate of 35.49% (Fig. 3B). Increasing salt concentration gradually reduced the phosphate-solubilizing capacity of B4. The strain grew rapidly and maintained strong solubilizing activity at salt concentrations between 0% and 5%, but its capacity decreased markedly at a salt concentration of 5% (Fig. 3C). In inorganic phosphorus liquid medium, ions such as Mg^2+^, K^+^, and Ca^2+^ significantly enhanced the phosphate-solubilizing ability of B4, yielding solubilization rates of 32.91%, 29.96%, and 29.83%, respectively (Fig. 3D). In contrast, Cu^2+^ addition resulted in the lowest level of phosphorus activation, though the solubilization rate still reached 8.93%. Notably, at a salt concentration of 7%, the activated phosphorus amount was 0.363 mg/L, demonstrating that strain B4 possesses considerable salt tolerance.

### *Advenella alkanexedens* alters soil nutrient profiles and enzyme activities

**Fig 4:**
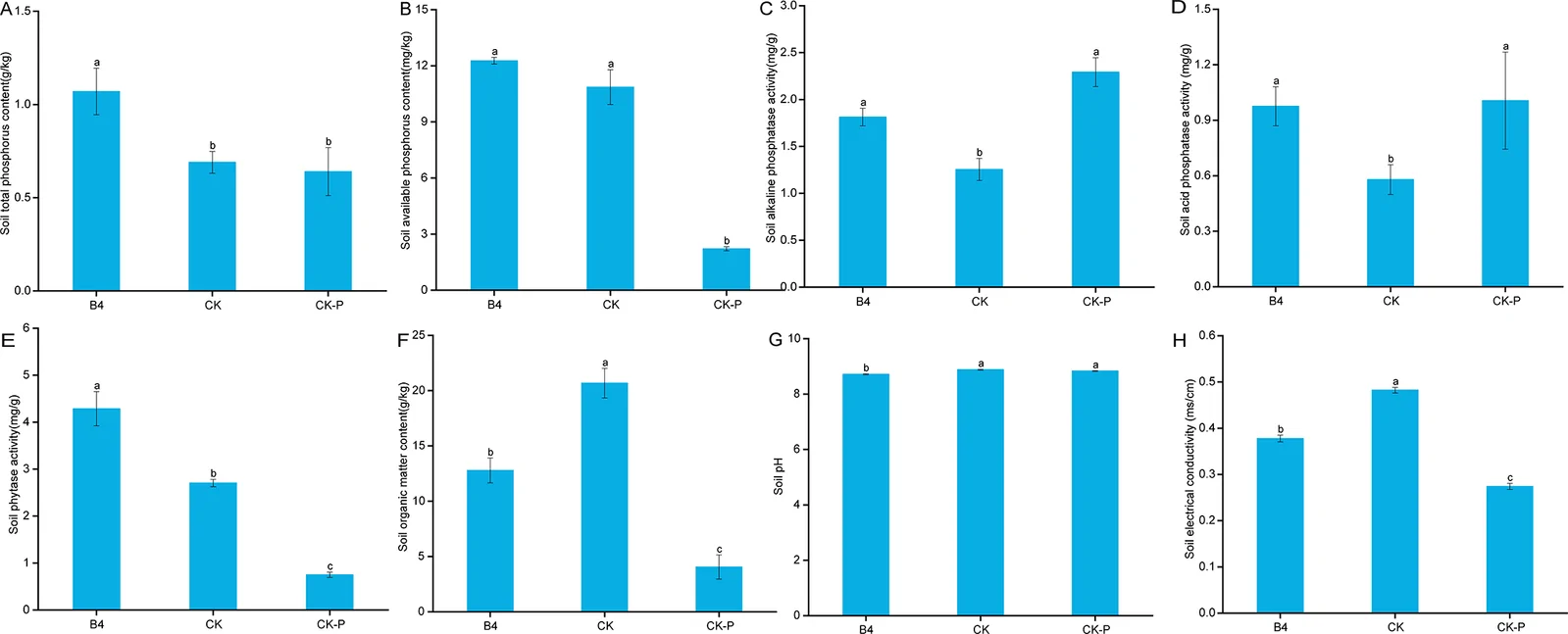
Physical and chemical properties of wheat soil under different treatments. (**A**) total phosphorus, (**B**) available phosphorus, (**C**) alkaline phosphatase, (**D**) acid phosphatase activity, (**E**) phytase activity, (**F**) organic matter, (**G**) pH, and (**H**) electrical conductivity. Different lowercase letters indicate significant differences before and after returning to soil in different treatments (*P* < 0.05).

The different treatments positively influenced soil nutrient levels and enzyme activities (Fig. 4). Soil organic matter (SOM) and electrical conductivity (EC) were higher in the control group (CK) than in the CK-P and B4 treatments, while the B4 treatment significantly increased both parameters relative to CK-P. The application of strain B4 compensated for phosphate deficiency and enhanced total phosphorus (TP) and organic matter content in the soil. Both soil available phosphorus (AP) and phytase activity (Phy) reached their highest levels under the B4 treatment, showing respective increases of 11.49%–81.91% and 36.87%–82.49%. Soil acid phosphatase (ACP) and alkaline phosphatase (ALP) activities followed the order CK-P > B4 > CK, with no significant difference observed between CK-P and B4. Soil pH decreased significantly in the B4 treatment, whereas no significant difference was found between CK and CK-P. These results demonstrate that the application of B4 bacterial solution can improve TP, AP, and PhyP in the soil.

### Shifts in soil inorganic phosphorus fractions mediated by *Advenella alkanexedens*

**Fig 5:**
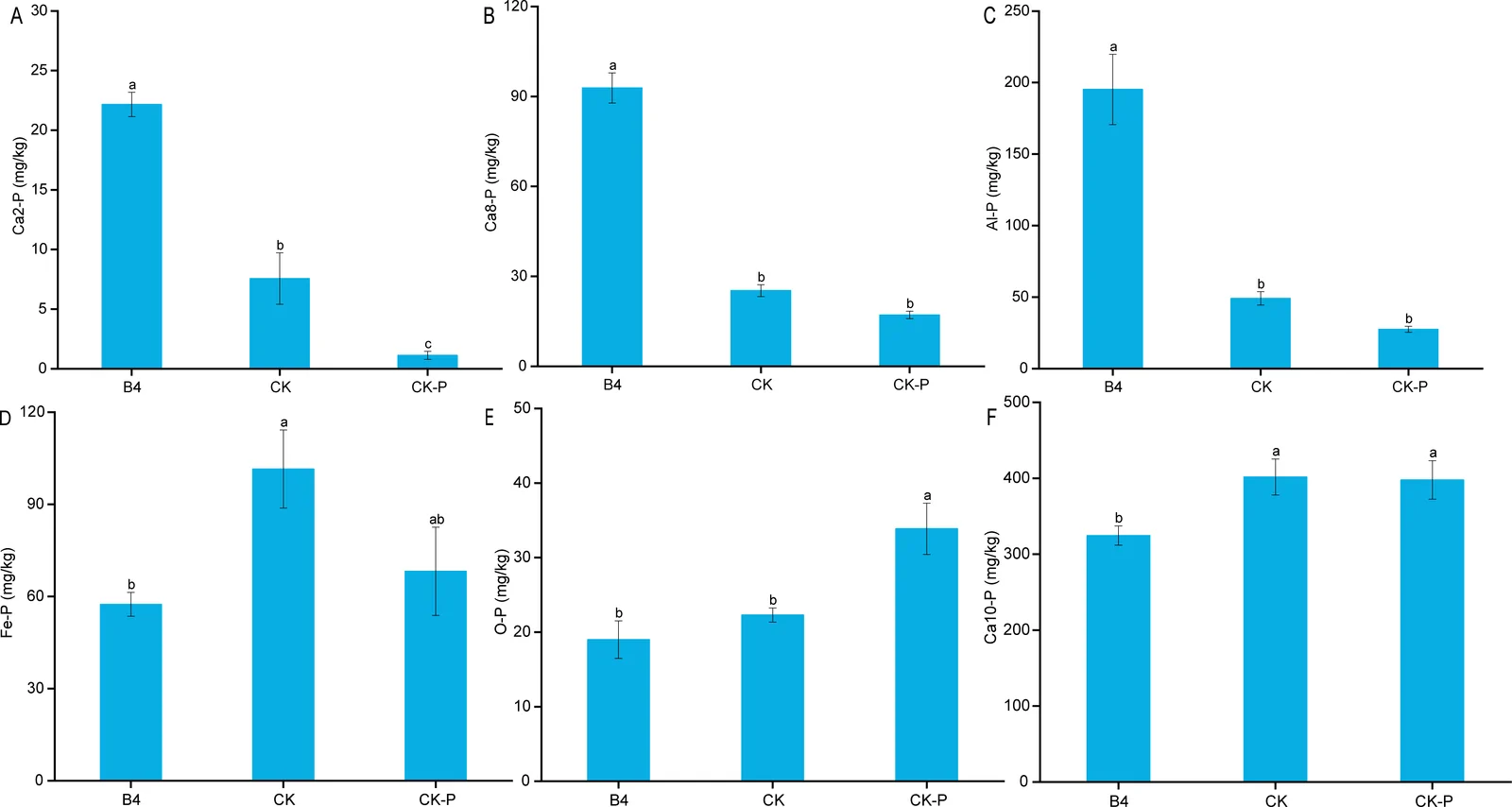
Changes of inorganic phosphorus content in wheat soil under different treatments. (**A**) Ca2-P, (**B**) Ca8-P, (**C**) Al-P, (**D**) Fe-P, (**E**) O-P, and (**F**) Ca10-P. Different lowercase letters indicate significant differences in different treatments (*P* < 0.05).

Soil inorganic phosphorus content was significantly altered by the different treatments (Fig. 5). Under the B4 treatment, the concentrations of Ca_2_–P, Ca_8_–P, and Al–P were significantly higher than those in the CK and CK-P control groups. Specifically, Ca_2_–P content increased by 65.84%–94.91%, Ca_8_–P by 70.54%–81.59%, and Al–P by 74.87%–80.81%. In contrast, Fe–P and Ca_10_–P contents showed a consistent trend across the three treatments, with the highest levels in CK, followed by CK-P, and the lowest in B4. Overall, the B4 treatment enhanced the proportion of active inorganic phosphorus fractions and most substantially reduced the content of insoluble phosphorus components.

**Fig 6:**
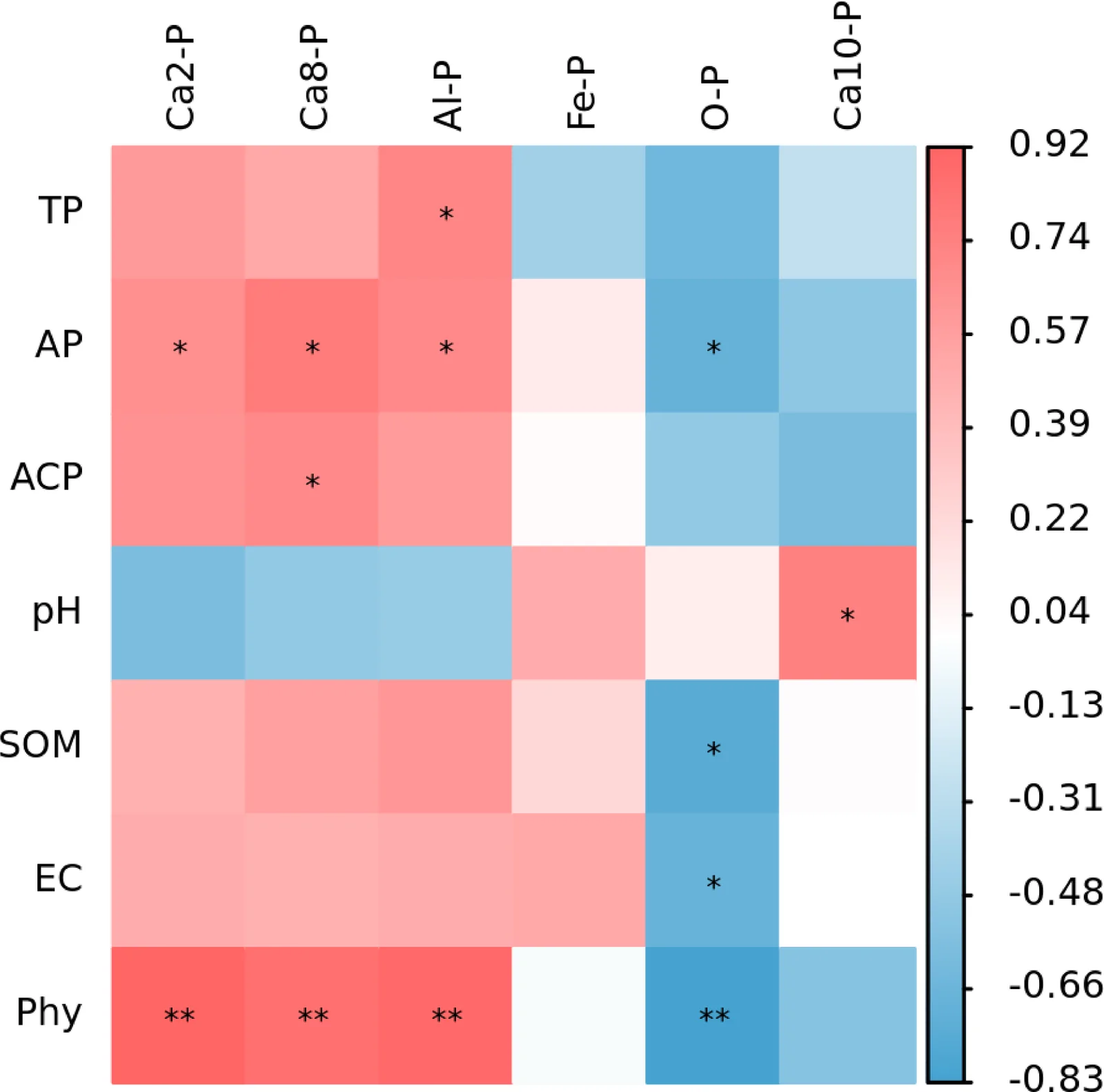
Pearson correlation analysis between soil inorganic phosphorus components and soil physical and chemical properties (*P ≤ 0.05, **P ≤ 0.01).

The B4 treatment showed the highest total inorganic phosphorus content in soil. Following wheat cultivation, the soil phosphorus activation coefficient increased (Table 2), and B4 consistently demonstrated effective phosphate-solubilizing activity throughout the trial. Pearson correlation analysis revealed that soil physicochemical properties differentially influenced the various phosphorus fractions (Fig. 6). Specifically, Ca_2_–P, Ca_8_–P, and Al–P were positively correlated with soil AP and Phy, while O–P was negatively correlated with AP, SOM, EC, and Phy.

**TABLE 2:** Inorganic phosphorus content and phosphorus activation coefficient in soil of wheat seedling stage^*a*^

	Total inorganic phosphorus (mg/kg)	Proportion of InP (%)	PAC (%)
B4	711.31 a	0.70 b	1.21 a
CK	607.37 b	0.88 a	1.62 a
CK-P	545.56 c	0.85 a	0.35 b

^*a*^ InP, inorganic phosphorus; PAC, phosphorus activation coefficient = AP/TP × 100%; different lowercase letters in the same column indicate significant differences between treatments (*P *< 0.05).

### Decoding the metabolome of *Advenella alkanexedens*: insights from fermentation broth analysis

#### Composition of metabolites

**Fig 7:**
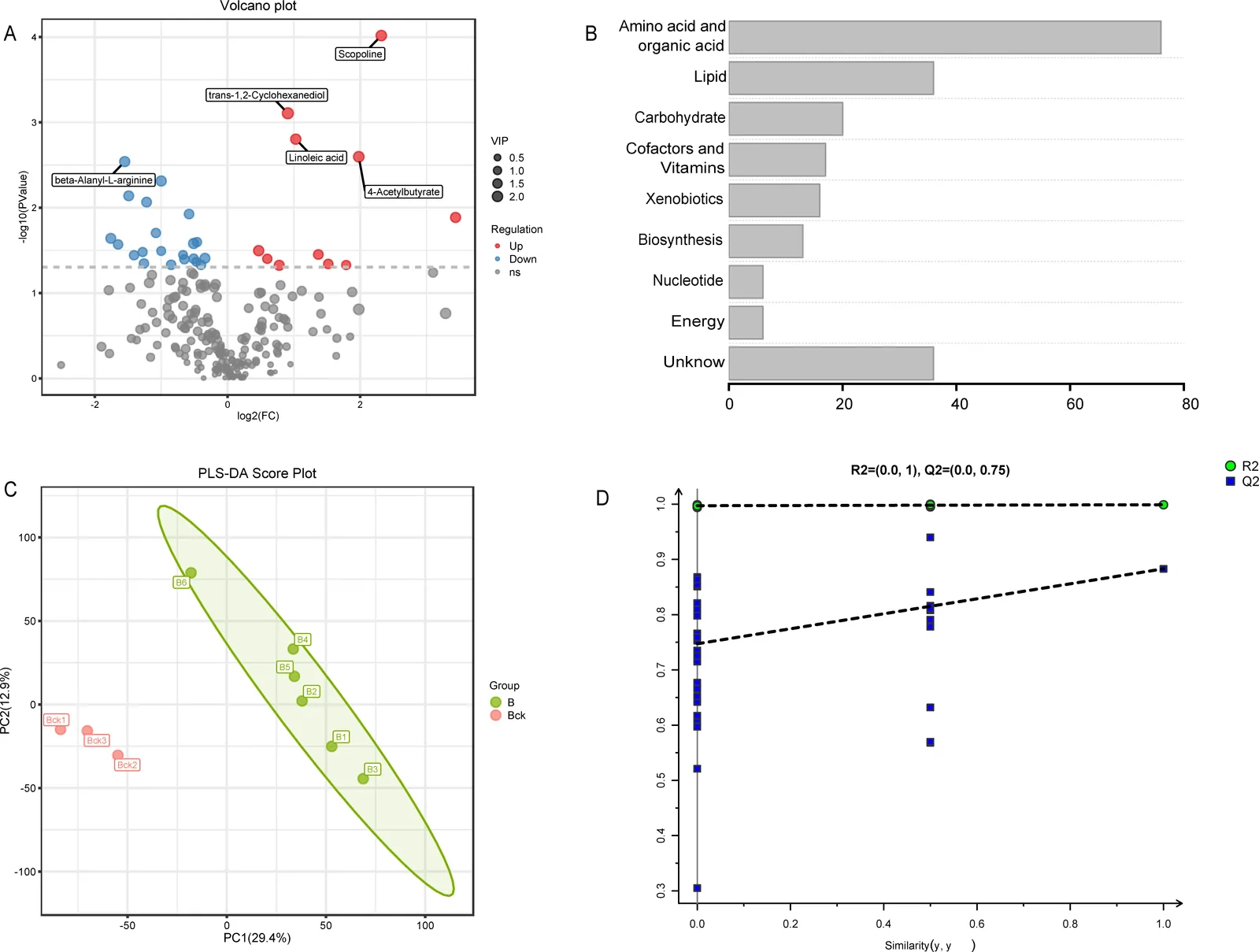
Composition of *Advenella alkanexedens* microbial metabolites. Volcanic plot of differential metabolites (**A**); the metabolites identified in all samples and the types and quantities of compounds to which these metabolites belong (**B**); PLS-DA score plots of phosphate-solubilizing bacteria metabolites (**C**); (PLS-DA) permutation test (**D**).

To investigate the metabolite profile of *Advenella alkanexedens* and its role in phosphorus activation, we conducted a whole-targeted metabolomics analysis of its fermentation broth, with reference to soil available phosphorus, inorganic phosphorus fractions, and phosphorus activation coefficient. A total of 226 metabolites were detected across all samples, including 125 unique metabolites. Compared with the blank control (without bacterial fermentation), 32 differential metabolites were identified, of which 11 were up-regulated and 21 were down-regulated (Fig. 7A). Cluster heatmap analysis revealed a distinct metabolite profile in the phosphate-solubilizing bacterial broth relative to the blank medium. These metabolites were structurally classified into 76 acids (amino acids and organic acids), 36 lipids, 20 carbohydrates, 17 cofactors and vitamins, 16 xenobiotics, 13 biosynthesis intermediates, 6 nucleotides, 6 energy-related compounds, and 36 metabolites of unknown origin (Fig. 7B). Using PLS-DA, we confirmed 32 differential metabolites. The PLS-DA results further demonstrated clear separation between metabolite groups upon introduction of the phosphate-solubilizing bacterium (Fig. 7C and D).

#### Differential metabolites

**Fig 8:**
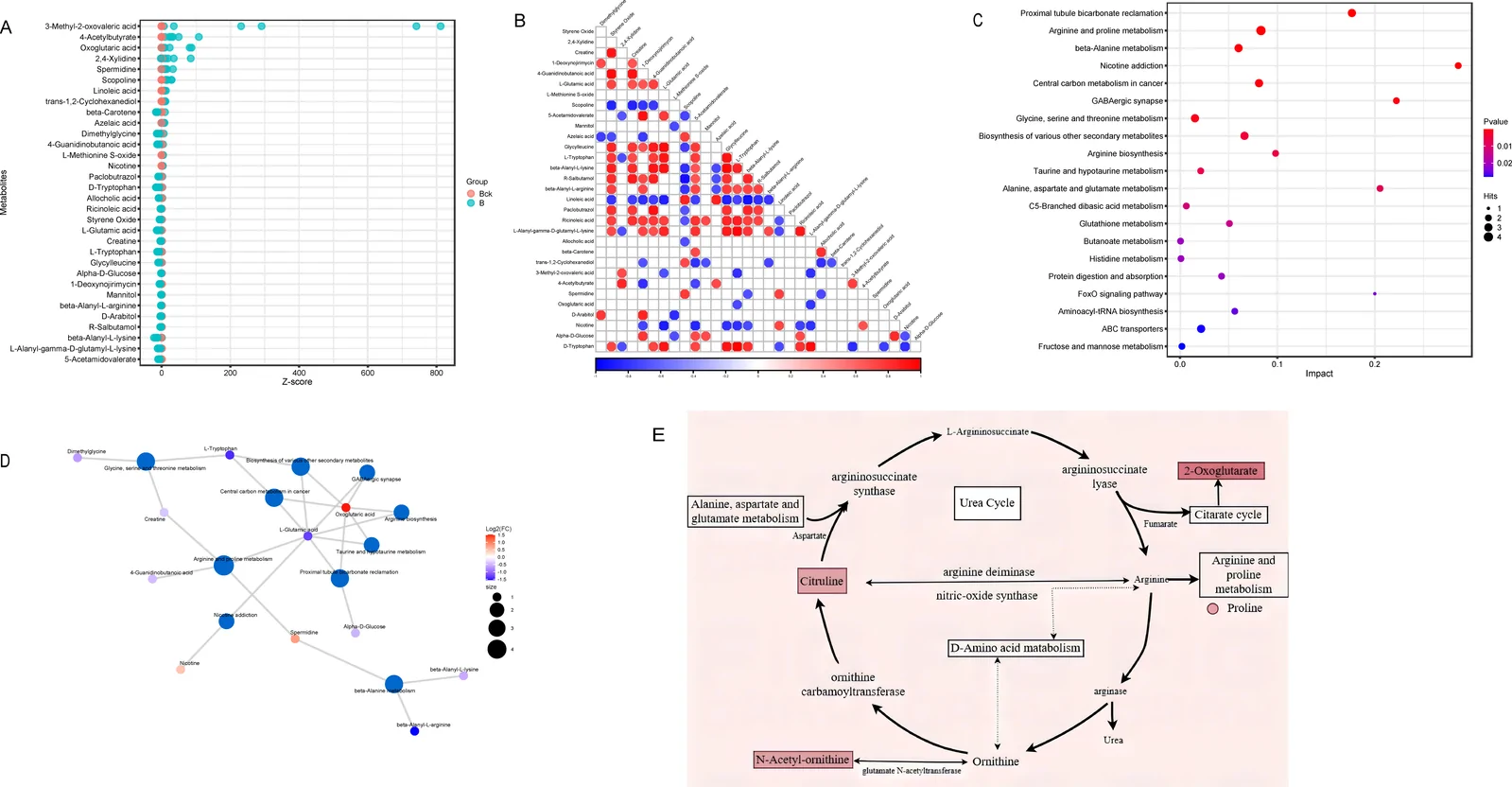
Z-score of differential metabolites (**A**); correlation thermogram of differential metabolite (**B**); bubble diagram of metabolic pathway influencing factors (**C**); the abscissa is the impact value enriched in different metabolic pathways, and the ordinate is the enrichment pathway. The point size indicates the number of corresponding metabolites in the pathway. Color is related to *P* value; the redder the color, the smaller the *P* value, and the bluer the color, the larger the *P* value. Metabolic pathway network diagram (**D**); the main pathways involved in the secretion of organic acids and amino acid metabolites by *Advenella alkanexedens* (**E**); red means up, blue means down.

As shown in Fig. 8, the levels of several metabolites—including 3-methyl-2-oxovaleric acid, 4-acetylbutyrate, oxoglutaric acid, 2,4-xylidine, spermidine, scopoline, linoleic acid, and trans-1,2-cycloxanediol—were elevated in the phosphate-solubilizing bacterial fermentation broth relative to the control (Fig. 8A). A significant positive correlation was observed among 2,4-xylidine, 3-methyl-2-oxovaleric acid, and 4-acetylbutyrate (Fig. 8B). These results suggest that *Advenella alkanexedens* can enhance phosphorus mobilization in the wheat rhizosphere through the secretion of acidic metabolites. KEGG pathways enrichment analysis further indicated that *Advenella alkanexedens* was predominantly associated with proximal tubular bicarbonate reclamation, arginine and proline metabolism, and beta-alanine metabolism (Fig. 8C).

Guanidinoacetate kinase and glycocyamine kinase act as phosphotransferases, utilizing nitrogenous groups as acceptors to transfer phosphate groups. Guanidinoacetate kinase participates directly in arginine metabolism and indirectly influences proline synthesis. Both the arginine/proline and β-alanine metabolic pathways enhance β-alanine production by up-regulating spermidine dehydrogenase (Fig. 8D).

#### The pathway analysis of key metabolites: organic acids and amino acids

Metabolomic analysis of *Advenella alkanexedens* (Fig. 8E) revealed that the up-regulation of citrulline was associated with enhanced activity in the citrate cycle (TCA cycle), arginine and proline metabolism, and ornithine metabolism. This metabolic shift also led to increased levels of proline and N-acetylornithine. Collectively, these findings indicate that *Advenella alkanexedens* improves phosphorus activation capacity by stimulating the citrate cycle, thereby promoting the dissolution of insoluble phosphorus fractions.

**Fig 9:**
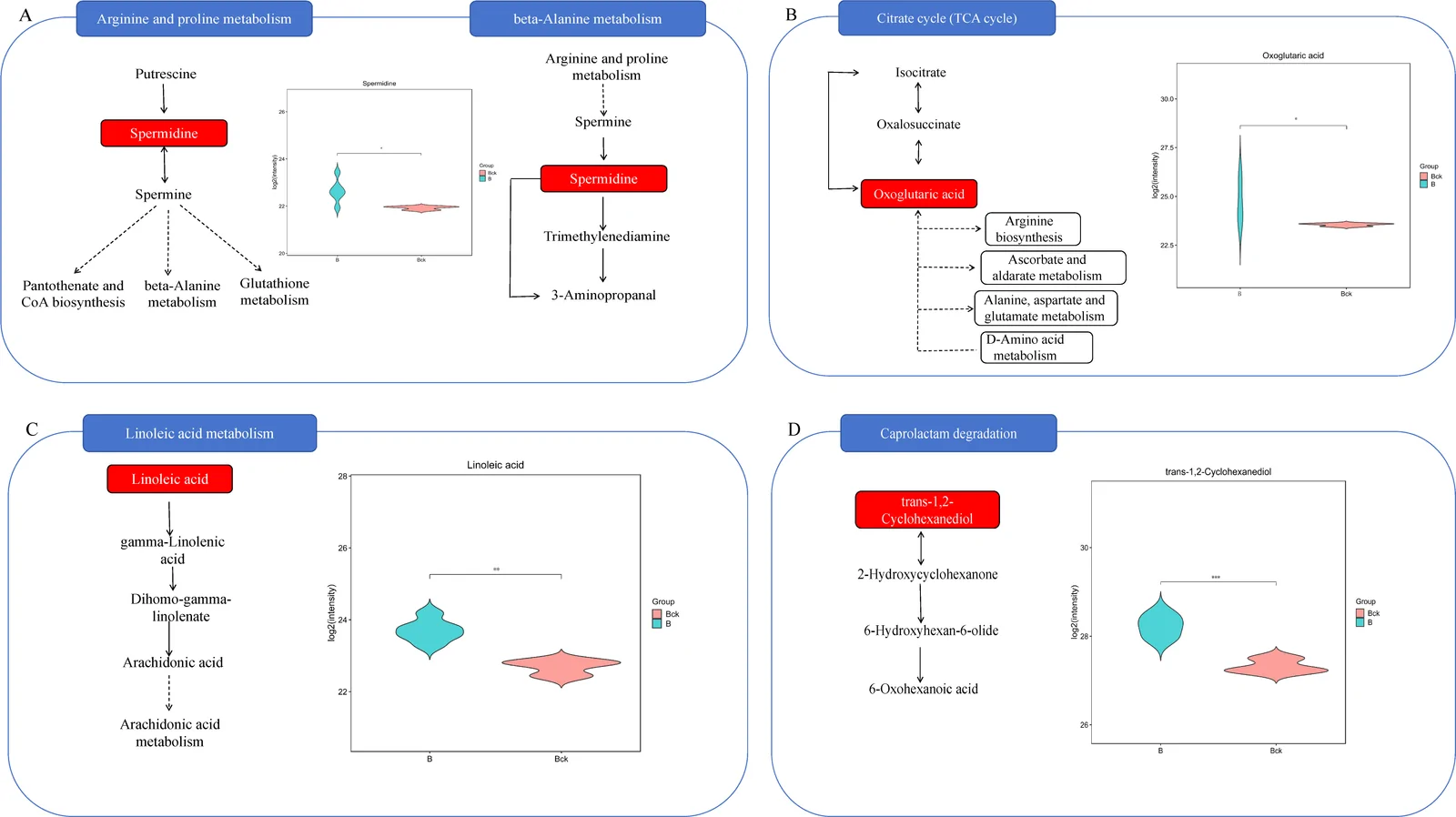
B4 significantly up-regulated the metabolic pathways of different differential metabolites. (**A**) arginine and proline metabolism and beta-alanine metabolism, (**B**) citrate cycle (TCA cycle), (**C**) linoleic acid metabolism, and (**D**) caprolactam degradation.

Based on the bubble diagram of metabolic pathways and the analysis of differential metabolites (Fig. 8A and C), five metabolic pathways linked to the phosphate-solubilizing capacity of the strain were identified: the citrate cycle (TCA cycle), arginine and proline metabolism, β-alanine metabolism, linoleic acid metabolism, and caprolactam degradation. As presented in Fig. 9, the up-regulated differential metabolites associated with these pathways included oxoglutaric acid (involved in the TCA cycle); spermidine (participating in both arginine/proline and β-alanine metabolism); linoleic acid (related to linoleic acid metabolism); and trans-1,2-cyclohexanediol (associated with caprolactam degradation).

## DISCUSSION

In the present study, an *in vitro* screening was performed using dominant microorganisms isolated from rapeseed rhizosphere soil. Their phosphate-solubilizing ability was first evaluated on NBRIP medium. Combined with pot trials, strain B4, showing the strongest wheat-affiliated phosphate-solubilizing effect, was ultimately selected. Morphological observation and 16S rRNA gene sequencing identified strain B4 as *Advenella alkanexedens*. To date, PSB have been mainly reported in genera such as *Bacillus*, *Pseudomonas,* and *Burkholderia* (33). As a plant-growth-promoting bacterium and phosphate-solubilizing bacterium, *Advenella alkanexedens* has been isolated from diverse environments, including biogas sludge, wild animals' intestines, and plant rhizosphere (21,34). Members of the genus *Advenella* exhibit versatile metabolic traits; for instance, *A. kashmirensis* is a facultative chemolithoautotroph, and *A. mimigardefordensis* can degrade polythioesters. This genus thus shows considerable potential in bioremediation, xenobiotic degradation, and biotechnological applications (34). As an environmentally benign bacterium with multiple biological functions, *Advenella alkanexedens* was further investigated in this study. We optimized its growth conditions, clarified its plant-growth-promoting traits, and elucidated the potential mechanisms underlying its phosphate-solubilizing ability.

The *Advenella alkanexedens* isolated in this study functioned as a PSB, capable of mobilizing soil phosphorus. The P-solubilizing capacity of PSBs is influenced not only by their intrinsic traits but also by external environmental factors. Previous studies indicate that the diversity and functionality of PSBs are closely associated with abiotic conditions, such as pH, temperature, nutrient stoichiometry, and anthropogenic activities (34, 35). Cultivation-based observations further show that factors, including soil pH, temperature, metal ions, salinity, and alkalinity, can affect the metabolic activity, enzyme secretion, and population dynamics of PSBs, thereby modulating their P-solubilization efficiency (36). Strain B4 exhibits effective phosphorus activation at room temperature, with its highest solubilization efficiency reaching 40.78%, a level sufficient to facilitate crop uptake of insoluble phosphorus. However, its activity is markedly inhibited at temperatures below 20°C, a pattern consistent with observations in other phosphorus-solubilizing bacteria (34, 37). In this study, phosphorus activation was strongest at pH 7 and declined under more acidic or alkaline conditions. This aligns with the known chemistry of phosphorus in soil: below pH 5.5, P tends to precipitate with Al^3+^ and Fe^3+^, while above pH 7, it forms precipitates with Ca^2+^ (38). Furthermore, the presence of metal ions significantly influenced solubilization. Adding K^+^, Mg^2+^, or Ca^2+^ to the medium enhanced the phosphorus-solubilizing capacity of B4, a result consistent with the findings of Dat et al. (39). Strain B4 demonstrated phosphorus-solubilizing activity even at 7% NaCl, indicating good salt tolerance. This is consistent with reports that many PSBs exhibit strong salt adaptability. For example, *A. kashmirensis* strain IB-K1 alleviates salt stress in moderately saline soil (40), and some PSB strains can grow at NaCl concentrations up to 20% (41, 42). These findings collectively suggest a close link between salt tolerance and phosphorus-solubilizing function. Our results confirm that environmental factors interact with PSB physiology to regulate phosphorus mobilization. Understanding this bio-ecological relationship will help select suitable PSB strains for different agricultural systems (43).

Phosphorus deficiency severely limits crop production, as phosphorus is essential for plant physiological processes and overall growth (44). Using wheat as the test crop, this study evaluated the effects of the phosphate-solubilizing *Advenella alkanexedens* under low-P stress. We found that its bacterial suspension significantly promoted wheat seedlings’ growth. Furthermore, application of B4 bacterial solution improved root and leaf quality, plant height, and stem-leaf phosphorus content to levels comparable to standard fertilization (Table S2). These results align with previous reports that PSMs can partly replace chemical P fertilizers under P-deficient conditions. Boroumand et al. (45) reported that *Pseudomonas stutzeri* and *Mesorhizobium* sp. enhanced the growth of land cress by increasing soil nitrogen and phosphorus availability. Borgi et al. (46) reported that *Serratia plymuthica* BMA1 increased the dry weight of inoculated *Vicia faba* L. plants by 76% and raised root and shoot phosphorus content threefold compared to non-inoculated controls. Additionally, Wang et al. (47) found that inoculating *Paenibacillus mucilaginosus* alleviated phosphorus stress in *Poncirus trifoliata* seedlings and enhanced plant growth along with nitrogen and phosphorus uptake. These studies collectively highlight the strong potential of PSMs to substitute for mineral phosphorus fertilizers. Our results further indicate that strain B4 promotes wheat growth under phosphorus-deficient conditions primarily by mobilizing the native soil phosphorus pool to meet plant demand, rather than by improving the efficiency of externally applied fertilizer. Moreover, combining PSMs with different phosphorus sources and nutrients, such as iron and silicon, has been shown to effectively increase crop phosphorus uptake and use efficiency, thereby supporting growth and yield (48). Galeano et al. (49) recently observed that inoculation with phosphate-solubilizing *Trichoderma* strains significantly increased shoot biomass, chlorophyll and phenolic content, and antioxidant activity in soybean under both fertilized and unfertilized conditions, while allowing for reduced fertilizer inputs without compromising yield. Together, these findings underscore the value of synergistic interactions between PSMs and reduced phosphate fertilization in enhancing phosphorus-use efficiency and mitigating environmental risks.

However, a limitation of this study was the absence of a combined “B4+reduced phosphate fertilizer” treatment, which prevented verification of any synergistic effect between the strain and lower fertilizer inputs. Future work will examine the performance of a B4-based microbial fertilizer in combination with chemical fertilizers. By testing graded reductions in phosphate application, we aim to clarify the strain’s mechanism of action under varying low-phosphorus regimes, thereby providing a clearer theoretical basis for implementing “reduced input, enhanced efficiency” strategies in agriculture.

This study found that B4 application increased soil phosphorus content, which aligns with recent research demonstrating that PSB inoculation significantly enhances soil phosphorus levels. For example, Xing et al. (50) reported that PSMs notably increased the TP content in plant roots. Microbial inoculation also markedly affected rhizosphere soil enzyme activities. In the absence of live inoculation, ALP and ACP activities were higher under phosphorus-deficient conditions compared to phosphorus-sufficient conditions. This likely reflects increased root exudation of organic acids, amino acids, and carbohydrates under P-deficient conditions (51), a result consistent with the present study. P-deficient soil thus triggers plants to upregulate nutrient acquisition mechanisms, including specific enzymes (52). Similarly, Bao et al. (53) observed that phosphate-solubilizing *Pyrenochaetopsis tabarestanensis* strains increased phosphorus availability by 5.80%–7.70% in rice soils, highlighting the role of PSB in improving soil fertility. Chatterjee et al. (54) further showed that PSB inoculation increased ALP activity in rice paddies, promoting phosphorus turnover. Furthermore, PSB can enhance the activities of other enzymes involved in carbon cycling, such as β-glucosidase and cellulase (55). These findings indicate that PSB not only improves phosphorus availability but also stimulates broader soil biochemical activity. The soil used in this study had an initial pH of 8.17, with calcium phosphate, Ca_3_(PO_4_)_2_, serving as the phosphorus source. Inoculation with the B4 strain significantly lowered soil pH, suggesting the secretion of acidic substances. This acidification enhanced soil enzyme activity and facilitated the conversion of insoluble phosphorus into soluble forms. In conclusion, PSB plays a crucial role in elevating soil phosphorus levels and stimulating enzyme activity, thereby supporting soil health and crop productivity. Recent studies reinforce their potential in sustainable agriculture, particularly in phosphorus-deficient soils. Future work should aim to optimize PSB formulations and elucidate their interactions with other soil microorganisms to maximize agronomic benefits.

The composition of soil inorganic phosphorus varies with parent materials, pH environment, and cropping conditions, which collectively influence phosphorus bioavailability and the soil supply capacity (56). Different chemical forms of phosphorus can undergo (57), while phosphorus often combines with metals, such as Ca, Fe, and Al, to form mineral precipitates or becomes adsorbed onto iron/aluminum (hydroxide) oxides, leading to P fixation in soil (58). Many bacteria secrete organic acids, including carboxylic acids, which can increase the solubility of calcium phosphate (59) and facilitate the dissolution of insoluble inorganic phosphate, such as tricalcium phosphate, dicalcium phosphate, hydroxyapatite, and phosphate rock, thereby enhancing phosphate fertilizer efficiency (60, 61). In our study, the application of PSB significantly activated various inorganic phosphorus fractions, most notably the highly plant-available Ca_2_–P fraction (62). This effect is attributed to PSB-secreted organic acids that solubilize calcium-bound phosphates (63). Among these, citric acid shows the highest solubilization efficiency for Ca–P (64), and the B4 strain was found to up-regulate differential metabolites associated with the TCA cycle. We also observed a substantial increase in Al–P fractions, consistent with reports that PSB can solubilize iron and aluminum phosphates via chelation and reduction mechanisms (65). These results align with earlier findings that PSB can raise phosphorus availability by 20%–40% across soil types (66). Notably, different phosphorus fractions exhibit distinct solubilization pathways: Ca–P is the most readily dissolved by organic acids; Al–P requires stronger organic acids, such as oxalic acid; and Fe–P depends on combined chelation and reduction processes (67). The differential activation of phosphorus fractions observed here provides mechanistic insight into phosphorus solubilization and highlights the potential of PSB as a sustainable alternative to chemical phosphorus fertilizers in agricultural systems.

PSB employs multiple physiological mechanisms to solubilize phosphorus, including acidolysis, enzymatic mineralization, hydrolysis of insoluble phosphates, and chelation or complexation of soil cations. During growth, PSB secretes various low-molecular-weight organic acids, such as malic, succinic, oxalic, and glutamic acid, which, under acid conditions, chelate metal ions (Ca^2+^, Fe^2+^, Al^3+^) via their hydroxyl and carboxyl groups, thereby releasing bound phosphorus (5, 68). In this study, the B4 increased soil phosphatase and phytase activities, indicating its ability to produce these enzymes, release protons, and promote phosphorus activation (65, 69). Metabolite analysis revealed that amino acids, organic acids, and lipids were the predominant metabolites associated with PSB-mediated phosphorus transformation. Notably, aliphatic acids demonstrated greater phosphate-solubilizing efficiency than phenolic, citric, or fumaric acids, supporting earlier findings by Kalayu (70). Furthermore, genetic studies have linked phosphorus solubilization in bacteria, such as *Bacillus megaterium,* to genes involved in organic acid synthesis (71). Collectively, these results underscore that organic acid secretion is the principal mechanism through which PSB enhances phosphorus availability in soil.

Pathway enrichment analysis indicated that metabolites influencing the phosphorus-solubilizing ability of B4 were primarily enriched in amino acid metabolism, specifically arginine and proline metabolism, and beta-alanine metabolism. As a key intermediate in the TCA cycle of PSB, oxoglutaric acid releases protons (H^+^) upon secretion, lowering the pH in the micro-environments and enhancing the dissolution of insoluble phosphates (e.g., calcium, iron, and aluminum phosphate). Furthermore, oxoglutaric acid chelates metal ions, such as Ca^2+^, Fe^3+^, and Al^3+^, disrupting phosphate mineral structures and increasing plant-available inorganic phosphorus (Pi), a mechanism particularly important in alkaline and calcareous soils (72, 73). The marked up-regulation of spermidine activated arginine-proline and beta-alanine metabolic pathways. Spermidine, an important regulatory molecule in bacterial cells, promotes organic acid secretion by regulating PSB-related genes (e.g., *gcd* and *pqq)* (15). Moreover, its polyamine structure enables metal ion chelation and proton release (74).

Activation of the linoleic acid metabolic pathway represents a key adaptation of PSB to low-phosphorus stress. Under phosphorus limitation, genes linked to linoleic acid metabolism are upregulated, driving acetyl-CoA production via β-oxidation. Acetyl-CoA enters the TCA cycle, boosting synthesis of potent organic acids, such as citric acid and α-ketoglutaric acid. Linoleic acid often works synergistically with other metabolites like spermidine and α-ketoglutaric acid, forming a multi-mechanistic network that enhances phosphorus solubilization. For instance, acetyl-CoA from linoleic acid metabolism can promote spermidine biosynthesis, which further stimulates organic acid secretion through the arginine-proline pathway (75). Additionally, trans-1,2-cyclohexanediol reduces phosphorus-metal binding affinity, releasing free phosphate ions. It improves phosphate solubility under acidic conditions—especially for calcium phosphates in alkaline soils—and increases acid/alkaline phosphatase activities, thereby promoting organic phosphorus hydrolysis and replenishing the inorganic phosphorus pool (33, 61). These findings align with the conclusions of Xia et al. (76).

### Conclusion

As a natural reservoir of soil phosphorus, PSB have been developed into eco-friendly functional biofertilizers. Their application offers a promising direction for green and sustainable crop production, with the potential to reduce dependence on conventional chemical fertilization and alleviate environmental pressure. In this study, a phosphate-solubilizing strain identified as *Advenella alkanexedens* was isolated for the first time from the rhizosphere soil of rapeseed. Application of this strain (B4) at the wheat seedling stage significantly enhanced soil phosphorus availability. PSB can mobilize insoluble inorganic phosphorus in calcareous soil, overcoming the limited phosphorus accessibility typical in such environments. The improved phosphorus supply directly promoted wheat seedling stage growth, establishing a foundation for the subsequent crop yield formation. Metabolomic analysis identified key metabolites associated with this strain, primarily comprising amino acids and organic acids. KEGG pathways enrichment further revealed that significantly up-regulated differential metabolites, such as spermidine, involved in arginine-proline and β-alanine metabolism, and oxoglutarate participating in the citrate cycle, collectively underpin the molecular mechanism of enhanced phosphorus solubilization. Thus, this study not only physiologically confirms the role of strain B4 in increasing soil available phosphorus but also mechanistically elucidates its metabolic basis for phosphorus activation. These findings provide a theoretical foundation for the efficient development and optimized application of PSB-based biofertilizers.

However, this study has some limitations. The controlled and homogeneous conditions of pot culture cannot fully replicate the complex natural environment of the field, which may cause the experimental outcomes to differ from actual field performance. In subsequent work, we will focus on developing biofertilizers derived from strain B4, including the selection of suitable carriers and optimization of bacterial survival and stability. Field trials will also be conducted to verify the practical efficacy of B4, thereby further enhancing its application potential.

## Data Availability

The data supporting the conclusions of this article are included within the article. The sequencing data have been uploaded to the NCBI database (accession number: PV576239).

## SUPPLEMENTAL MATERIAL

Fig. S1; Tables S1 to S4 spectrum.03481-25-s0001.docx Screening of strains for pot application.

ASM does not own the copyrights to Supplemental Material that may be linked to, or accessed through, an article. The authors have granted ASM a non-exclusive, world-wide license to publish the Supplemental Material files. Please contact the corresponding author directly for reuse.
