# Supplementary material for: Advenella alkanexedens, a specific phosphate-solubilizing bacterium from rapeseed rhizosphere soil, highly activates insoluble phosphorus in calcareous soil
Source: Microbiol Spectr. 2026 May 26;14(7):e03481-25. doi: 10.1128/spectrum.03481-25 (PMC13340305; doi:10.1128/spectrum.03481-25)
Supplement: Fig. S1; Tables S1 to S4 — Screening of strains for pot application. [file spectrum.03481-25-s0001.docx]

**Appendix**

**
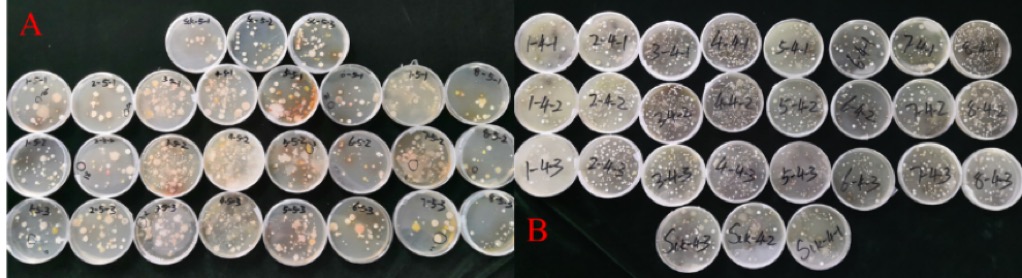
**

Figure S1 Culturable dominant bacteria(A) and fungi(B) in the rhizosphere soil of rapeseed

Note：The culture plate number does not represent the treatment number

Table S1 Functional Analysis of *Advenella alkanexedens* Strain

| Nitrogen  fixation | Dissolution of organic phosphorus | Dissolution of inorganic phosphorus | Potassium dissolving | Produce ACC  deaminase | Siderophore |
| --- | --- | --- | --- | --- | --- |
| - | - | + | - | - | + |

Table S2 Effects of different microbial solution on agronomic characters at wheat seedling stage

| Number | Root fresh weight(g) | Root dry weight(g) | Fresh weight of stem and leaf(g) | Dry weight of stem and leaf(g) | Plant height(cm) |
| --- | --- | --- | --- | --- | --- |
| B1 | 0.42±0.03cd | 0.05±0.02a | 0.54±0.07g | 0.07±0.01fg | 17.10±0.35ef |
| B2 | 0.41±0.03cd | 0.07±0.00a | 0.75±0.05defg | 0.09±0.01def | 19.07±0.47abc |
| B3 | 0.46±0.03cd | 0.05±0.00a | 1.05±0.04abc | 0.13±0.01abc | 19.40±0.31ab |
| B4 | 0.87±0.18a | 0.05±0.01a | 0.96±0.12abcde | 0.12±0.02abcde | 20.10±0.86a |
| F1 | 0.80±0.11ab | 0.07±0.01a | 0.99±0.03abcd | 0.12±0.00abcde | 19.97±0.62a |
| F2 | 0.47±0.10cd | 0.06±0.01a | 0.73±0.09efg | 0.09±0.01def | 17.77±0.15cde |
| F3 | 0.37±0.02d | 0.04±0.01b | 0.80±0.03def | 0.10±0.01cde | 19.23±0.53ab |
| F4 | 0.63±0.12abcd | 0.05±0.01a | 0.80±0.10def | 0.10±0.02cde | 19.97±0.54a |
| F5 | 0.53±0.14bcd | 0.06±0.02a | 0.82±0.04cdef | 0.11±0.00bcde | 18.77±0.18abcd |
| F6 | 0.52±0.04bcd | 0.04±0.00b | 1.07±0.07ab | 0.15±0.01ab | 19.23±0.24ab |
| F7 | 0.63±0.14abcd | 0.07±0.01a | 0.89±0.06abcde | 0.12±0.00abcde | 17.43±0.24def |
| F8 | 0.40±0.05cd | 0.04±0.01b | 0.9±0.01abcde | 0.11±0.00bcde | 19.67±0.22ab |
| F9 | 0.71±0.16abc | 0.05±0.01a | 1.12±0.16a | 0.15±0.02a | 19.43±0.43ab |
| F10 | 0.39±0.06cd | 0.05±0.01a | 0.63±0.05fg | 0.08±0.01efg | 17.50±0.38def |
| CK | 0.37±0.02d | 0.03±0.00b | 0.87±0.05bcdef | 0.11±0.01bcde | 18.37±0.45bcde |
| CK-P | 0.38±0.01d | 0.05±0.00a | 0.29±0.02h | 0.06±0.00g | 16.13±0.52f |

Table S3 Effects of different microbial solution on plant phosphorus and soil nutrients at wheat seedling stage

| Number | Root phosphorus content (g/kg) | Phosphorus content in stems and leaves (g/kg) | Soil total phosphorus content (g/kg) | Soil available phosphorus content (mg/kg) | Soil alkaline phosphatase activity (mg/g) | Soil organic matter content (g/kg) |
| --- | --- | --- | --- | --- | --- | --- |
| B1 | 7.08±0.23a | 4.95±0.07abc | 0.75±0.05abcd | 10.07±0.66abc | 0.55±0.02d | 12.05±2.93bcd |
| B2 | 3.68±0.50c | 5.54±0.04abc | 0.51±0.14cd | 8.52±0.82abc | 1.77±0.14ab | 8.73±1.83bcd |
| B3 | 5.02±0.50abc | 4.85±0.68abc | 0.62±0.12abcd | 7.37±0.39bc | 0.96±0.09bcd | 16.83±1.97abcd |
| B4 | 4.94±0.13abc | 5.35±0.37abc | 1.02±0.23a | 12.27±0.18a | 1.81±0.09ab | 12.78±1.12bcd |
| F1 | 4.81±0.79bc | 5.84±0.14ab | 0.84±0.09abcd | 11.05±0.45abc | 0.57±0.14d | 6.86±0.18bcd |
| F2 | 5.97±0.92ab | 3.78±0.71c | 0.60±0.04bcd | 7.29±0.51bc | 1.73±0.20abc | 19.43±3.79abcd |
| F3 | 4.39±0.35bc | 4.92±0.40abc | 0.70±0.01abcd | 12.21±0.64a | 1.31±0.02bcd | 19.95±2.21abc |
| F4 | 4.61±1.19bc | 5.70±0.09ab | 0.98±0.25ab | 11.56±0.71ab | 1.80±0.25ab | 16.52±3.43abcd |
| F5 | 3.94±0.48bc | 4.15±0.23bc | 0.49±0.09d | 10.46±1.01abc | 0.99±0.14bcd | 15.28±2.95abcd |
| F6 | 4.63±0.36bc | 5.27±0.51abc | 0.86±0.06abcd | 8.09±1.49abc | 1.25±0.08bcd | 30.76±2.00a |
| F7 | 3.51±0.97c | 6.24±0.57a | 0.76±0.23abcd | 10.90±0.60abc | 0.87±0.17cd | 3.33±0.55d |
| F8 | 5.15±0.53abc | 4.32±0.58bc | 0.92±0.03abc | 7.50±0.60bc | 0.49±0.02d | 15.69±2.52abcd |
| F9 | 5.18±0.42abc | 4.51±0.09abc | 0.59±0.04bcd | 7.83±0.99abc | 1.07±0.12bcd | 17.35±1.87abcd |
| F10 | 4.43±1.17bc | 4.12±0.08bc | 0.56±0.02cd | 6.66±0.96c | 0.73±0.02d | 20.47±1.71abc |
| CK | 5.95±0.06ab | 4.25±0.33bc | 0.67±0.12abcd | 10.86±0.93abc | 1.25±0.12bcd | 20.68±1.34ab |
| CK-P | 3.18±0.29c | 0.59±0.13d | 0.64±0.04abcd | 2.22±0.11d | 2.29±0.15a | 4.05±1.09cd |

Table S4 Principal component analysis of growth promotion effect of different microbial solution at wheat seedling stage

| Number | Principal component score | | | | Comprehensive score | Ranking |
| --- | --- | --- | --- | --- | --- | --- |
|  | F1 | F2 | F3 | F4 |  |  |
| B1 | -2.07 | 1.51 | -0.18 | -1.49 | -0.59 | 15 |
| B2 | -0.37 | -0.52 | 1.03 | 0.45 | -0.01 | 9 |
| B3 | 0.83 | -0.41 | -0.43 | -0.55 | 0.09 | 7 |
| B4 | 0.91 | 1.27 | 0.89 | 1.49 | 0.69 | 1 |
| F1 | 0.65 | 0.83 | 2.14 | -0.86 | 0.48 | 2 |
| F2 | -0.73 | -0.29 | -0.09 | 0.4 | -0.23 | 13 |
| F3 | 0.03 | 0.43 | -1.25 | 0.24 | -0.06 | 10 |
| F4 | 0.29 | 1.14 | 0.08 | 1.14 | 0.36 | 4 |
| F5 | 0.22 | -0.76 | 0.49 | -0.25 | 0 | 8 |
| F6 | 1.75 | -0.37 | -1.53 | -0.01 | 0.28 | 5 |
| F7 | -0.05 | 0.1 | 2.14 | -0.92 | 0.17 | 6 |
| F8 | 0.18 | 0.38 | -1.07 | -0.89 | -0.12 | 11 |
| F9 | 1.5 | -0.64 | 0.19 | -0.06 | 0.38 | 3 |
| F10 | -0.82 | -0.88 | -0.65 | -0.86 | -0.53 | 14 |
| CK | -0.15 | 0.58 | -1.89 | 0.04 | -0.2 | 12 |
| CK-P | -2.19 | -2.35 | 0.13 | 2.14 | -0.73 | 16 |
